# Optimizing brain mapping: integrating real-time neuropsychological assessment in awake craniotomy

**DOI:** 10.3171/2024.10.FOCVID24128

**Published:** 2025-01-01

**Authors:** Neslihan Nisa Gecici, Ahmed Habib, Ajay Niranjan, Jeffrey Balzer, Natalie Sherry, Pascal O. Zinn

**Affiliations:** 1 Department of Neurological Surgery, University of Pittsburgh School of Medicine, Pittsburgh, Pennsylvania; and; 2 Department of Neurological Surgery, University of Pittsburgh Medical Center, Pittsburgh, Pennsylvania

**Keywords:** brain mapping, awake craniotomy, neuropsychology, cognitive

## Abstract

Intraoperative neuropsychological testing (IONT) is a sophisticated method of cognitive mapping during the resection of brain tumors in eloquent areas. Direct electrical stimulation during awake craniotomy is routinely utilized for mapping basic language and sensorimotor function, but the utilization of IONT offers an individualized approach that can yield real-time, comprehensive feedback on various cognitive functions, allowing for a tailored and more extensive tumor resection. In this video, the authors present the case of a 41-year-old male undergoing re-resection for a recurrent right temporal astrocytoma in which IONT played a crucial role.

The video can be found here: https://stream.cadmore.media/r10.3171/2024.10.FOCVID24128

## Transcript

### 0:20

In this video, we are going to discuss the role of intraoperative neuropsychological testing, a novel approach offering continuous information about a myriad of cognitive functions. Direct electrical stimulation, or DES, mapping is the go-to technique for identifying essential brain functions during surgery.^1,2^ This method has been invaluable in minimizing postoperative deficits while simultaneously allowing for the removal of as much of the tumor as possible—key for improving patient survival. However, while its use is established for simpler tasks, such as sensorimotor functions or basic language exercises in dominant hemisphere tumors, there is increasing focus on the preservation of higher-level cognitive functions, especially in subcortical regions or during surgeries in the nondominant hemisphere.^3^

### 1:05

When it comes to intraoperative neuropsychological testing, or IONT, we are looking at a major leap forward in how we monitor brain function during surgery. It offers a continuous stream of information on how the brain is functioning as we remove the tumor. It is analogous to having a real-time conversation with the brain—where we can monitor language, visual-spatial judgment, neglect, attention, and even higher-order executive functions all at once.^4^ It also reflects the functioning of broader neural networks, providing a dynamic view of brain function as opposed to the circumscribed focus on specific language hubs.^4^

### 1:42

Real-time intraoperative neuropsychological testing at our program utilizes an advanced dual iPad application setup, where one iPad is positioned for the patient to view and interact with the tasks, while the other is used by the neuropsychologist to monitor and control the process.^5^ The examiner’s iPad is equipped with the ability to provide an auditory signal to cue the neurosurgeon for stimulation, which is crucial because if the stimulation and presentation of the stimulus do not coincide, it could lead to incorrect interpretations. This setup also captures patient accuracy, reaction time for response, and audio and video recordings of the patient, giving us comprehensive data to analyze later. The app also supports multiple testing paradigms, rendering it a versatile tool for assessments during resections in areas with the potential of multiple eloquent functions.

### 2:31

In our intraoperative neuropsychological testing, we selected several tasks to utilize during mapping and neuromonitoring, including verbal and nonverbal tasks. We select the tasks based on several factors, including tumor location, baseline neuropsychological test data, patient symptoms, and the cognitive skills most vital to the quality of life of our patient.

### 2:53

We utilized an object naming task for mapping in this case as it is considered the gold standard for assessing language function and requires perceptual object recognition.^6^ This paradigm also effectively targets core language areas in the brain and is both simple and efficient. Moreover, its strong standardization and adaptability make it a reliable choice across different clinical settings. To better illustrate the impact of IONT, we will be presenting a case where this approach played a crucial role.

### 3:20

This case involves a 41-year-old right-handed male with a recurrent *IDH*-mutant, *ATRX*-mutated right temporal astrocytoma. He initially underwent resection of the low-grade astrocytoma in 2016. Over the years, he has experienced episodes of dizziness, a rising sensation of hot flashes, and panic attacks that occur unexpectedly. In May 2021, MR imaging showed progression of the right temporal lesion. He then underwent near-total resection of the nonenhancing tumor. Following surgery, he received radiation therapy with concurrent temozolomide and completed 12 cycles. He denied cognitive deficits and was working full-time in a technical computer science field since the prior surgery. Recent MRIs revealed a mass-like hyperintensity in the right insula and a small enhancing nodule in the posterior insula, suggesting a high-grade transformation. As a result, we recommended re-resection.

### 4:16

Before surgery, we first localized the somatosensory, motor, and language areas in the brain using magnetoencephalography, or MEG. To assess the language areas, we used two specific paradigms. Firstly, we had the patient listen to five target words. Then, whenever one of those words was repeated, the patient was asked to raise a finger if they recognized it. The second paradigm involved two tasks: object naming and word reading. These tasks were designed to engage both language perception and production areas of the brain. For the object naming task, the patient was shown pictures of objects and asked to quickly think of the noun that describes the object. After the picture, a word was shown, and the patient had to decide whether the word matched the picture. This task helped us identify language production areas, like Broca’s area, and also areas involved in lexical information about the language, like Wernicke’s area. The word reading task, on the other hand, activated a wider network of language-related areas responsible for recognizing words, accessing their meanings, and integrating them into a coherent understanding. Interestingly, the MEG results showed bilateral activation in language-related areas, with stronger activation on the right side during the word listening task. This finding was unusual, especially since the patient is right-handed. Because of the possibility that critical language areas might be in the right hemisphere and the demand on nonverbal functions to his profession, we decided to move forward with a redo awake craniotomy, including continuous real-time neuropsychological testing, to ensure the patient’s safety.

### 5:50

In our preoperative neuropsychological assessment, our goal is to set a solid baseline to guide everything in the operating room. The patient completes a traditional comprehensive neuropsychological evaluation to objectively measure global cognitive performance, as well as baseline testing to subsequently use in the operating room. The comprehensive neuropsychological preoperative assessment revealed that the patient is very high functioning, with most of his scores well above average for his age group. There were no signs of cognitive impairment or language deficits, consistent with his self-report. However, we did notice a relative weakness in visual-based learning and memory compared to verbal memory and other verbal-based functions. Based on this discrepancy, the neuropsychological test data supported that the lesion was likely located in the nondominant hemisphere and/or impacting nonverbal functions. The absence of cognitive impairment was also a good prognostic indicator that the tumor had not yet affected the crucial functional networks in the brain.

### 6:49

In terms of obtaining a baseline for use in intraoperative mapping, we looked at a few key areas: First, we assess standard language tasks, like object naming, auditory comprehension, and word reading. This helps us understand the patient’s core language abilities. Next, we check functions related to the nondominant hemisphere. This includes tasks like line bisection and number span, which help us spot any issues with neglect or spatial orientation. We also consider the patient’s quality of life. For instance, our patient was working in IT, and we included tasks related to math calculations to see how well he can handle tasks important to his job. For intraoperative testing, a 70% of accuracy is recommended as a cutoff.^7^ The items that the patient answered correctly were selected for use in the intraoperative testing; those that were wrong were excluded.

### 7:46

Here, you see the intraoperative footage showing the real-time neuropsychological assessment in action and how it helps us neurosurgeons to maximize the extent of resection safely and preserving the patient function. The surgeon gives the stimulation after hearing the beep signal from the iPad and maps the brain based on the continuous feedback from the patient.

### 8:07

[Tasks are performed during surgery to assess the patient’s response during brain mapping.]

### 9:34

Intraoperatively, we had positive stimulation despite the tumor being in the nondominant hemisphere, as suggested by MEG. During the mapping session, while resecting the tumor, we administered over 70 object naming trials to the patient. We recorded 4 perceptual errors and 3 instances of mild dysarthria. In this image from the neuronavigation, you can see the point corresponding to all 4 perceptual errors we encountered intraoperatively. Integrating intraoperative neuropsychological testing with the surgery helped us find the safest margin for resection while ensuring the preservation of function.

### 10:11

The patient tolerated the procedure well and was taken to the ICU. Subsequently, he was transferred to the floor in stable condition. His postoperative course was unremarkable. He had no neurological deficits or complications. Postoperative imaging revealed postoperative changes following right temporal craniotomy and mass resection. There were no findings to suggest residual tumor. The patient was discharged on POD 1. At 2-week postoperative neuropsychological screening, the patient was evaluated for gross cognitive and language dysfunction. He did remarkably well and had no deficits. He will undergo repeat comprehensive neuropsychological evaluation at 6 months postoperatively.

### 10:50

In summary, IONT provides continuous cognitive monitoring during the resection of tumors located in eloquent areas and maximizes the preservation of functions and extent of resection.

## Disclosures

The authors report no conflict of interest concerning the materials or methods used in this study or the findings specified in this publication.

## Author Contributions

Primary surgeon: Zinn. Editing and drafting the video and abstract: Zinn, Gecici, Habib, Sherry. Critically revising the work: all authors. Reviewed submitted version of the work: Gecici, Habib, Niranjan, Balzer, Sherry. Approved the final version of the work on behalf of all authors: Zinn. Supervision: Habib, Niranjan, Sherry. Attending neuropsychologist: Sherry.

## Supplemental Information

### Patient Informed Consent

The necessary patient informed consent was obtained in this study.
